# The COVID-19 emergency as an opportunity to co-produce an innovative approach to health services provision: the women's antenatal classes move on the web

**DOI:** 10.1007/s43039-021-00045-6

**Published:** 2022-02-05

**Authors:** Manila Bonciani, Ilaria Corazza, Sabina De Rosis

**Affiliations:** Management and Healthcare Laboratory (MeS Lab), Institute of Management, EMbeDS, Sant’Anna School of Advanced Studies, Pisa, Italy

**Keywords:** Healthcare services provision, Value co-creation, e-health/m-health, Antenatal classes, Maternal care pathway, COVID-19 pandemic, action research

## Abstract

The COVID-19 pandemic has strongly affected healthcare organizations, leading to the need for reorganizing also maternal care services during pregnancy. The Regional Health Authorities in Tuscany (Italy) promoted the creation of online antenatal classes (ACs). This study illustrates the innovative approach to deliver ACs online and discusses how the collaborative approach in co-producing this innovative solution co-creates value in healthcare. The action research design was based, on one hand, on the indirect involvement of users by analyzing qualitative data collected through a continuous survey to pregnant women and, on the other one, on the direct involvement of managers and health professionals in meetings and workshops. The authors encompassed all necessary changes in organizational practices and facilitated the collaborative process implementation and analysis. The main findings are that moving ACs online has been a relevant choice, since the need of pregnant women to share information and receive emotional support increased in times of crisis. Additionally, in the perspective of health professionals, the new online ACs model emerged as a valuable solution not only for the contingent situation, but also in a long-term perspective to reach more women during pregnancy and to early support them throughout the maternal care pathway. This study shows that the collaborative approach to co-innovate healthcare services provision, such as with ACs online, facilitates the creation of, long-lasting, and integrated solutions in healthcare, to be used also after pandemic period. Finally, despite this action-research is context-specific, the findings presented in this paper may help other healthcare organizations innovate their own strategies in ACs’ provision.

## Introduction

The COVID-19 pandemic has strongly impacted healthcare organizations in Italy, not only for the direct burden of case management and preventive measures, but also for the need to reorganize the provision of all services, even those not directly affected by the pandemic itself.

Despite the difficulties that the situation has entailed, healthcare organizations have faced an unexpected opportunity to carry out transformations and urgent innovations (Berry, 2019) in the service provision model, which would have encountered much resistance in the pre-COVID-19 period (Kellermann & Jones, 2013).

The pandemic has been a productive time for collaborative efforts of practitioners, users and researchers together, for coping with the challenges emerged during these unprecedented times, but also for facing still-existing inadequacies. Before the emergency outbreak, the digitalization of healthcare faced multifactorial challenges (Hashiguchi, 2020), which appeared relaxed during the pandemic. An extraordinary number of innovative responses to crisis were quickly adopted (Woolliscroft, 2020). However, due to the general unpreparedness of healthcare systems, digital and innovative solutions have been not properly and effectively governed, producing a patchy scenario, with bottom-up, context-specific or local-dependent solutions (Petracca et al., 2020). As a consequence, widen inequalities could have been produced among individuals, patients, users (Tarricone and Rognone, 2020).

The role of the research could have been key to accompany and guide the identification of equitable and long-lasting innovative solutions for the provision of services, as well as for measuring the extent of the transformations and the effects they have had on the organization itself and on users. In particular, it is interesting to study if the collaborative efforts of practitioners and researchers could have been an enabling factor of health service innovation in response to the COVID-19 and in the new normal times, as hypothesized by De Rosis and Barsanti in the pre-COVID-19 era (2017).

In this context, value co-creation processes involving different actors can have facilitated both the creation and diffusion of an innovation culture, and the further involvement of both healthcare services’ providers and users in innovating healthcare services (Berry, 2019). The marketing literature have increasingly focused on value co-creation considering providers and users of services, in the healthcare industry too (Beirão et al., 2017; Sweeney et al., 2015; Vargo & Lusch, 2008), and on the role that co-creation can have in innovating healthcare (Joiner & Lusch, 2016; Patrício et al., 2019).

A specific area of interest on which to focus the research activity has been represented by the services related to the maternal care pathway, in particular the non-clinical ones aimed at promoting women's health during pregnancy. Indeed, because of the COVID-19 pandemic, the maternal care pathway’s group activities were suspended. This is the case of antenatal classes (ACs) provided by healthcare organizations. The experience of Tuscan Regional Health Authorities is significant in this field. Thanks to the collaboration and coordination of a research group of the Management and Healthcare Laboratory of the Sant’Anna School, Tuscany Health Authority promoted the creation of online ACs, as an integrated solution with another existing m-health tool for the maternal care pathway, the mobile app hAPPyMamma (Bonciani et al. 2021). Despite various attempts made in various European countries to move ACs online during the pandemic period, to our knowledge studies regarding the innovation of these health services provision are not present in the scientific literature.

Focusing on the specific context of the Tuscany Region, the present paper aims to (i) describe the innovative approach, adopted by healthcare organizations, to delivery ACs online; (ii) discuss how the collaborative approach in co-producing this innovative solution co-creates value in healthcare.

This research contributes to the literature in different ways. First, it confirms the relevance of the co-production processes in facilitating the innovation design, acceptance, and implementation. Second, it uses a multi-stakeholder methodology of action-research, so providing real-word evidence to support the hypothesis of the importance of collaborative processes. Third, it provides qualitative insights focusing on the healthcare industry during a critical period, such as the COVID-19 pandemic, so shedding light on how empirically a collaborative approach can support a structured and standardized design and implementation of innovations in healthcare.

In the following sections, we develop the theoretical background, describe the methodology, and provide the results of our action research, then discussing the main implications for marketing theory and practice.

## Theoretical background

### Determinants of innovation use

Innovating healthcare services appeared urgent already before the COVID-19 pandemic (Berry, 2019). Previous research on digitalization of healthcare shows that there are several determinants, both facilitators and barriers, of successful adoption of innovation in healthcare (Chowdhury et al., 2019; De Rosis & Barsanti, 2017; Kierkegaard, 2015). In the pre-COVID-19 era, multifactorial barriers, ranging from regulatory to the natural human inclination to resist change have ‘saved’ the healthcare sector from disruptive or transformative changes of innovation (Hendy & Barlow, 2012), particularly in the public healthcare sector.

At the technology-level, the literature highlighted issues related to the definition of what e-health, tele-health, tele-medicine, tele-care are; problems of target-population, with a general focus on improving the delivery of care or the access to care for niches of population or where distance is a critical factor, such as in rural areas or developing countries; highly context-specific solutions and low scalability; and scarce available evidence, also due to a limited number of successful cases or best-practices (Chowdhury et al., 2019; De Rosis & Barsanti, 2017). At the level of individuals, and mainly professionals, a key role has been played by the champion and his/her personal networks (Chowdhury et al., 2019; De Rosis & Barsanti, 2017). In the first phase of innovation adoption, champions are highly effective; while, moving beyond local contexts, the effectiveness of the champions varied (Hendy & Barlow, 2012). Moreover, the Technology Acceptance Model (TAM) refers to ‘perceived ease of use’ as the key element of acceptance of innovations by professionals (Venkatesh and Davis, 2000; Davis, 1989). Perceived usefulness, perceived ease of use, and habit are key determinants of the physicians’ intention to use innovations (Liew et al., 2020), also according to the Unified Theory of Acceptance (UTAUT and UTAUT2) (Venkatesh et al., 2011). At the level of the healthcare organization, key determinants are responsibility and legal issues; the need for multi-disciplinary teams; the need of evidence on the solutions’ effectiveness (Hendy & Barlow, 2012); the systemic nature of the innovation in healthcare (Chen et al., 2014). Scholars underlined the need for a participative approach to healthcare digitalization, as well as for a collaborative culture of innovation in order to enhance the integration of innovative solutions into the practice (De Rosis & Barsanti, 2017; Hendy & Barlow, 2012). Value is created through innovation in healthcare through enhanced coordination, resource integration and collaboration of all different stakeholders, particularly professionals and users, in the innovative solutions’ design and provision, according to the co-production approach (De Rosis & Nuti, 2018; Sebastiani & Anzivino, 2020).

In the new normal, among determinants of innovation adoption in healthcare, facilitators are reported more than barriers, with pre-Covid-19 barriers that seem to be less relevant (Bashshur et al., 2020). Suddenly, the pandemic provided an urgent reason to overcome all the issues: to contain the epidemic, avoid contamination, and implement social distancing. The ‘perceived usefulness’ has trumped ‘perceived ease of use’ during the pandemic (Bidmead & Marshall, 2020). Interestingly, a recent study showed that before the pandemic, the use of technologies in healthcare, such as the Electronic Medical Record (EMR) was significantly affected by the technology perception of physicians. On the contrary, after the pandemic in the ‘new normal’ condition, technology perception no longer has any significant impact on physician productivity though their intention to use technologies with a very weak direct impact on their productivity (Liew et al., 2020). In this latter research, it emerged that the physicians’ dynamic capabilities, meant as resilience or capacity to manage organizations, activities, and tasks in dynamic and uncertainty environments, strongly and directly impact physician productivity (Liew et al., 2020). In changing and critical circumstances, digitalization has been recognized as the safe way to maintain the service delivery, while protecting both patients and professionals from risks of infection. Extraordinarily, an impressive number of innovative solutions were fast developed during the pandemic (Woolliscroft, 2020). In this respect, the COVID-19 emergency period could be identified as a ‘strategic window’, according to Abell’s model (1978), or, in other words, as a chance or opportunity for healthcare organizations to improve and innovate services through a creative combination of resources, whereby exceptional value is generated (Hougaard, 2006). As numerous studies have already demonstrated, this complex historical period played an excellent revelatory role and turned into a special real-world laboratory to dig deeper into the opportunities offered by innovation, digitalization and technologies to sustain and improve healthcare services design and provision in times of crisis (see for example Belso-Martínez et al., 2020), especially with respect to people-centred and integrated care (Patrício et al., 2019). Indeed, due to the diffusion of Coronavirus contagion and more particularly for the sake of its containment, most health care services could not be performed in presence anymore in order to align with the emerged necessity of personal and social distancing. Hence, as outlined by Heinonen and Strandvik (2021), this era settled the opportunity to define the concept of “imposed service innovation”, as counter posed to the traditional view of services innovation as a primarily discretionary activity (Heinonen & Strandvik, 2021). However, these innovations were implanted in relatively unprepared healthcare systems, such as the Italian one (Bosa et al., 2021; Petracca et al., 2020). A lack of a shared and effective governance of the innovative solutions led to patchy implementations, so reproducing some pre-pandemic issues, such as context-specific or local-dependent solutions, or inequalities in the access to the digital care (Tarricone & Rognoni, 2020). Moreover, the ‘perceived usefulness’ could change in a post-crisis period, and it is not clear whether and how long suspension or relax of regulations or barriers for innovative solutions’ deployment would be maintained, in part or in total, with the risk to simply fall back to status quo ante (Bashshur et al., 2020).

### Co-creating value in healthcare

Over the last two decades, the relationship among and between service providers and users has changed, in particular due to a change in relationships, expectations and roles of people in respect to the providers (Prahalad & Ramaswamy, 2004, Etgar, 2008, Hienerth et al., 2014, Cui & Wu, 2016, Carlborg et al., 2014, Nguyen et al., 2018, Trenz et al., 2018).

This phenomenon has been defined in various ways, including co-production. In this research, the authors refer to a voluntary, explicit, and conscious process of value co-creation. According to the Service Dominant (SD) logic, each service is characterized by an intrinsic co-production process, given by the interaction or encounter between provider and user (Norman, 1991; Vargo & Lusch, 2008; Osborne et al., 2016; Gronroos, 2008, 2011; Prahalad & Ramaswamy, 2004). In addition, Osborne identified an explicit process of active and voluntary engagement of people (Osborne et al., 2016). In his perspective, co-production consists in creating the right conditions for all stakeholders to explicitly produce value for themselves and the community. According to the SD logic, the healthcare organizations should consider both individual users and healthcare workers as potential sources of knowledge, innovation, and value creation (Vargo & Lusch, 2008). The same public servants and services’ users should be consciously and voluntarily engaged in the service co-production (co-design, co-delivery, co-assessment, co-innovation) (Brandsen & Pestoff, 2006; Osborne et al., 2016).

To this end, the healthcare organizations should work in making the healthcare workers aware and empowered in their role of creating value in respect to the interaction with the healthcare services’ users (Sorrentino et al., 2015; De Rosis et al., 2020). Healthcare professionals oversee care delivery in healthcare sector, which is traditionally dominated by the «reverse hierarchy» organizational structure (Mosley, 2014). According to the model of individual professionalism and clinical autonomy (Baker & Denis, 2011; Mintzberg, 1989), key decisions on patients’ care are taken by physicians during visits, by assessing patients’ needs and prescribing treatments and care (Baker & Denis, 2011). Professionals’ role in determining day-by-day practices and cost of care is extremely relevant (Tjosvold & MacPherson, 1996). Moreover, evolutive as well as innovative changes to healthcare service highly depend on physician openness to adapt to new work processes (Liew et al., 2020). Public healthcare managers are responsible for organization and financial sustainability of healthcare services, as well as for their performance and outcomes. Given their unique role, the engagement of healthcare professionals is a keystone of quality improvement processes in healthcare (Nuti et al., 2016), as well as in healthcare services innovation (De Rosis et al., 2019).

Given the complexity of healthcare systems, engaging all stakeholders is crucial to properly meet users’ needs and to respond to the whole population (Gray et al., 2017; Nuti et al., 2016). To this respect, the engagement of healthcare services’ users is equally critical (De Rosis et al., 2019).

The healthcare services’ users can be engaged as potential partners in co-production processes, and their knowledge, expertise and abilities considered as assets (Karazivan et al., 2015; Voorberg et al., 2014; Batalden et al., 2016). In this context, the user evaluation of experience with healthcare services can co-create value by informing quality improvement actions, and services’ innovation. Co-assessment process can match the citizens’ point of view with the healthcare professionals’ one for creating value at different levels: individual, related to a specific group of stakeholders or collective/social (Nabatchi et al., 2017).

In this perspective, the process of value co-creation is led by a co-production process enabled by internal and external stakeholders’ collaboration and participation to a value-creation system (Normann, 2001; Sorrentino et al., 2015; De Rosis et al., 2019). This process encompasses the combination of several different and sometimes conflicting motivations of each stakeholders’ group, to co-produce the service and to create value (Ostrom, 1996). Under this logic, the partnership between public servants and citizens is a crucial aspect in the co-production process (Pestoff, 2012), for the sharing of assets, resources, and contributions and for innovating services (Osborne et al., 2016; Vargo et al., 2020). Thus, introduction and diffusion of organizational innovations as well as technological innovation in healthcare should consider all actors as change co-enablers and resource integrators (Vargo et al., 2020). Although some research shows that diverse barriers and facilitators of co-created activities may emerge in the interactions among stakeholders (Virlée et al., 2020), some others demonstrate that the social context influences massively their participation in services co-production and adherence (Osei-Frimpong et al., 2016, Osei-Frimpong et al., 2015). Previous studies have emphasized the role that the co-production can play in enhancing an effective design and implementation of innovative solutions in healthcare (De Rosis & Barsanti, 2017).

### Aims and framework of the study

Value co-creation processes can be used to innovate or adapt the service delivery, to better fit with users’ and providers’ needs, as well as to external pressures, as the pandemic has been. Indeed, this co-creation process can have particular value facing the COVID-19 outbreak, which became an opportunity for innovating the traditional health services provision severely limited by the pandemic. Given this premise, the research question this study aims to answer is: can a collaborative approach to innovation design and implementation allow creating a new valuable model of health services provision that can be used, not only to answer to the immediate needs due to pandemic, but also with a long-lasting and integrated perspective?

The hypothesis is that a key role can be played by the collaboration of different stakeholders, namely professionals and users, in a co-production process moderated and guided by researchers, who take advantage of the “strategic window” due to pandemic to promote innovation in health service provision. In this study, the authors adopted the value co-creation perspective, in order to study the collaborative process supporting an innovation in the provision of a specific service (ACs) in the healthcare services user journey in the maternal care. The study aims to point out whether this collaborative process, can bring to an integrated and sustainable model.

In particular, the authors supported healthcare professionals and women in the maternal care pathway in:

- The identification of the rooms for innovation in the pathway, by supporting the expression of needs and preferences;
- The evaluation of perceived usefulness, perceived ease of use of specific technological solutions, and related habits of professionals to be changed;
- The identification of equitable and long-lasting innovative solutions, to better responding to both providers and users’ needs during and after the crisis;
- The identification of indicators to measure the innovation’s and transformations’ impact.

These key points constitutes the framework that have supported the researchers in the action research implementation and in the interpretation of its results.

**Fig. 1:**
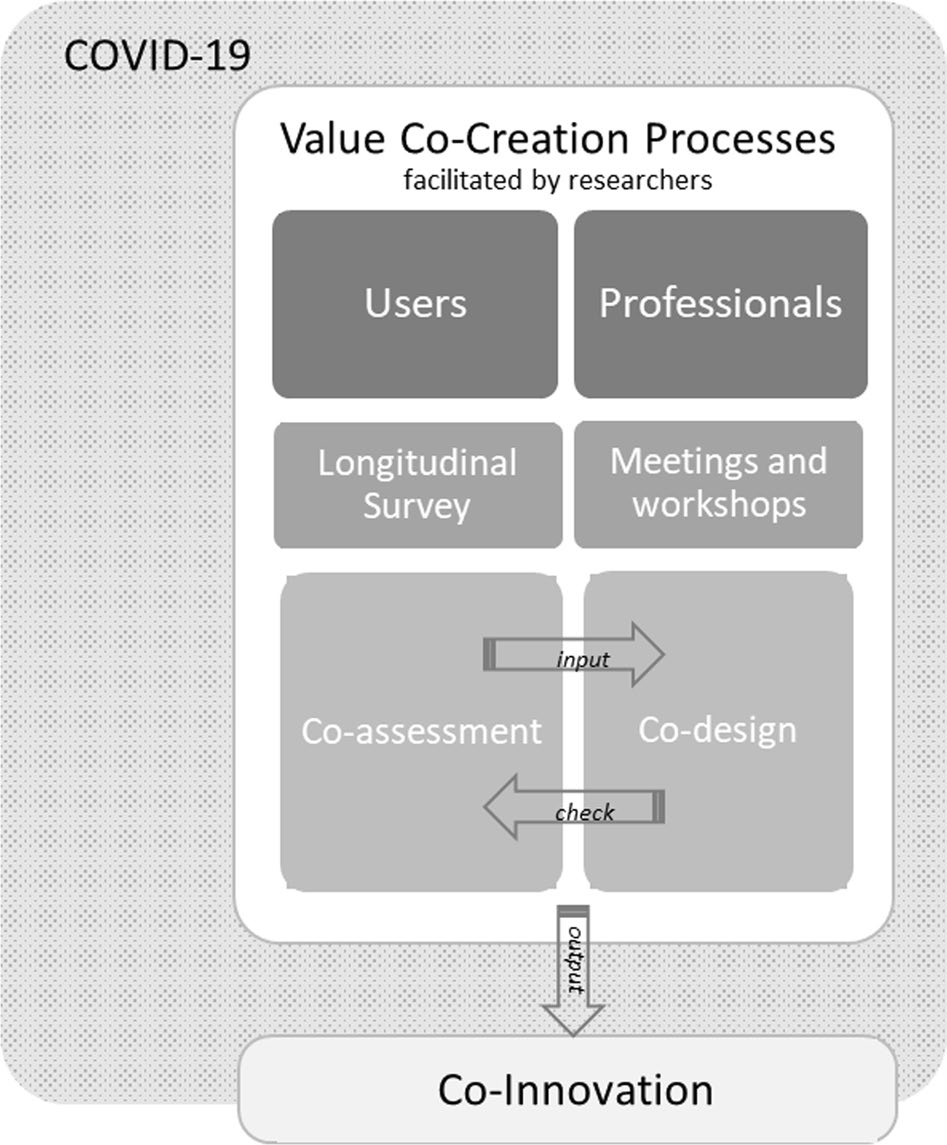
A model of the involvement of users and practitioners in an ACs’ co-assessment and co-design process

With this study, the authors investigate whether innovating the provision of a traditional maternal care service could be feasible by involving users and practitioners in an ACs’ co-assessment and co-design process. Figure 1 illustrates the over mentioned process.

As shown in Fig. 1, within the overall context of the COVID-19 pandemic, both pregnant women, as service users, and health professionals, as service providers, took part equally into the process of value co-creation (who is involved). The researchers, instead, facilitate mainly such a collaborative process and analyse whether and how the healthcare organizations could re-define services using users and professionals’ inputs. More particularly, pregnant women were involved indirectly, as the qualitative data from them were collected by means of an ongoing survey in Tuscany on the maternal and childcare pathway, while health professionals were involved directly, as they participated actively in project meetings and workshops (how the key actors are involved). Therefore, the contribution provided by pregnant women resulted mainly in the co-assessment of needs and requirements, and of their changes due to pandemic; health professionals contribute to the co-design process of the proposed solution aimed at mobilizing and integrating resources. Indeed, the researchers’ assumptions have been that:

- Users can actively contribute to the organization of services, by integrating fundamental resources for value creation (Zainuddin, 2013), such as information on their needs, preferences, and expectations.
- Health professionals, in turn, can integrate their competencies and skills and concretely change the service delivery, by participating as an active part of technological and organizational innovation.

The assessment provided by women triggered the necessity to design a tailored solution, whose design in turn offered the possibility to foster assessment of healthcare services. Such a cycle, facilitated by researchers throughout the various phases, resulted in the collaborative process of implementing the innovative solution.

## Methods

### Research design

This study is based on action research design applied to the specific context of the development of online ACs in Tuscany Region as an innovative approach to health services provision, made necessary by the COVID-19 outbreak. In particular, an iterative and cyclic protocol was adopted, in order to foster deeper understanding of the situation characterized by the suspension of face-to-face ACs and the need to remodel them online, starting with conceptualizing and specifying the problem and moving through the collaborative intervention to the online ACs design, implementation and analysis.

The action research approach is based on the collaboration between researchers and practitioners, which are respectively the research system and the practice system (Van de Ven & Johnson, 2006). This method has been largely used for service delivery development and for enabling a continued exploration of current issues in innovation management (IM), such as new organizing forms (Tanna, 2005; Ollila & Yström, 2020). This approach uses self-reflective spiral of cycles of observation, reflection and planning, implementation of action as the intervention, evaluation and moving to the next stage of planning. This way the research group is driven by questions originating in research based on explicit or implicit theories developed in previous research, but at the same time by problems originating in practice (Gummesson, 2000; Van de Ven & Johnson, 2006).

The present study is based on the action research developed within the real-world problem of ACs provision during the COVID-19 pandemic, encompassing changes in organizational practices and the direct participation of users and practitioners into the investigation and action. To address the critical situation of the ACs suspension due to the restrictions of the COVID-19 emergency, researchers involved directly or indirectly pregnant women and professionals of the maternal care pathway in a value co-creation process. This process was aimed at defining the problem (needs), identifying a solution (technology change) that is equitable and sustainable, implementing the proposed solution with the contribution of all actors and analysing what the new model of health services provision represents in a long-lasting and integrated perspective.

Since the innovation process of the ACs provision also involved the use of new digital tools, the researchers helped to create a vision around the digital innovation, supporting health professionals to see a reason to change and adapt their way of working and to establish a standardized digital model to promote a unified experience across healthcare operators and users. The consideration behind this value co-creation process initiated by the research group is that mobile healthcare applications can completely revamp healthcare processes based on the use and integration of e-communication at all levels and engender positive user experience and expectations (Lee, 2019).

### Context of application

The choice to focus on ACs represents a key example to analyse the collaborative value co-creation process in health care service, since it concerns health promotion activities where the involvement and participation of users is strategic for the achievement of the health objectives.

In general, this context of application is interesting for the relevance of the subject. Indeed, as affirmed by the World Health Organization, antenatal care is one of the most important services in health care and every pregnant woman should have full access to it (WHO, 2021). In fact, on one hand, antenatal care is associated with activities of prevention, identification, and treatment of “conditions that may threaten the health of the foetus or the new-born and, or the mother”, on the other, it helps women “approach pregnancy and birth as positive experiences” (WHO, 2021). Within antenatal care, ACs seem to improve women's knowledge and competence. This may provide a defence against the tendency to over medicalize pregnancy and childbirth (Spinelli et al., 2003).

Moreover, it is fundamental to mention the relevance of social roles and spaces in the peculiar domain of the maternal care pathway that is a life pathway rather than a care pathway. As such, women along the pathway can be considered as health and social services users rather than patients and they can play an important and active role for their health and that of their children. In particular, the ACs aims to sustain the women’s role as protagonist, by encouraging their empowerment and their maternal health literacy (Renkert & Nutbeam, 2001). In such a setting, the perceived value that this initiative may add to the pathway is crucial. Indeed, the positive experience of women in ACs and the perception of their usefulness may foster the possibility of women to take effectively benefits from attending ACs for their health and that of their children, as well as their fidelity toward the services provided by the public health system along the entire maternal care pathway. This perception of usefulness is guaranteed when ACs are built by adopting a flexible approach to accommodate participants' knowledge, goals, and preferences as well as characteristics of the context (Downer et al., 2020).

Finally, the choice of ACs as context of application of this study is relevant also for the role of health professionals who have a strong inter-relationship with women in this setting. This condition offers a great opportunity to observe how additional value for the pathway can be co-created by the collaboration of health professionals and users.

### Study setting

The present study was implemented in Tuscany Region where ACs are mainly organised by family care centres of the three Local Health Authorities (LHAs) as an important free-of-charge offer within maternal care pathway for health promotion and prevention of mothers and their children health. They are usually organised with quite small groups of pregnant women (15–20). They used to include several face-to-face meetings (generally from 6 to 10 meetings) almost in the final period of pregnancy, but also in post-partum, mainly with midwives, but involving also other professionals (gynaecologists, prevention doctors, paediatricians, psychologists, social workers). During ACs these professionals usually provide information to women on pregnancy, childbirth, breastfeeding, vaccination, children care and other themes relevant for mothers and their children health, but they focus also to create an interactive group where women may exchange experiences and emotions. ACs are organised also by the three regional Teaching Hospitals (THs) where women can have childbirth and receive second level assistance for high-risk pregnancies. In this setting, ACs can include larger groups of pregnant women and a less number of meetings, focusing mainly on childbirth assistance and breastfeeding, but they represent always an opportunity for women to acquire knowledge and develop skills in relation to the maternal care pathway. Due to the COVID-19 emergency, from March 2020 the ACs were suspended in order to avoid gathering of people in health facilities. During the following months characterized by lockdown and other restrictions, LHAs and THs organized themselves differently, at different times and not always homogeneously for all the territories, to continue to provide information and relational support to pregnant women.

The research group of the Management and Healthcare Laboratory of the Sant’Anna School, which have been already collaborating with the Regional Health Authority for the maternal care pathway monitoring and evaluation (Murante et al., 2014; Bonciani et al., 2020; Bonciani et al., 2021), proposed to coordinate an action-research intervention to cope with this criticality of the ACs interruption. The idea was to support the identification of a new model of ACs provision at the regional level thanks to the collaborative involvement of users and health professionals and enhancing the digital resources already present in Tuscany. Indeed, since March 2019 the Regional Health Authority promoted the implementation of hAPPyMamma system, which includes a mobile and web app for women and a web portal for operators, with specific but integrated functions for both (Bonciani et al., 2021). The app for users is a digital tool that accompanies women during pregnancy, childbirth and up to the child’s first year with the objective to facilitate the access to and use of the services and health opportunities of maternal care pathway. It provides to women:

- Personalized messages in the app home according to the pregnancy or postpartum period and display of upcoming appointments
- Information on the maternal care pathway, including multilingual, proactively proposed
- Georeferenced information on family care centers and birth hospitals and their services
- Possibility to make reservation for test and visits of the maternal care pathway
- Digital pregnancy booklet
- Possibility to participate to the systematic survey on the maternal care pathway proving feedback on their experience and perceived outcomes.

The web portal for health professionals of the hAPPyMamma system allows to manage the regional computerized archive of the pregnancy booklet records, the web prescription for the digital pregnancy booklet and the dynamic archive of information that are visualized in the mobile and web app. The research group started from the hypothesis to integrate in the hAPPyMamma system the new model of ACs, but it should have been co-created with users and professionals to be perceived as useful and effective for both.

### Data collection

The action research design included two phases addressing respectively users and health professionals. The first involved the analysis of the data collected by the continuous survey on the experience of users within the maternal care pathway. Researchers analysed and valued needs, wishes and experiences that pregnant women reported about ACs. Researchers used the narrative comments that women left in specific spaces of the questionnaire related to their experience with the ACs. The open question concerns specifically what positively or negatively affected women about the AC they have attended. The questionnaire is administered digitally. The questions on ACs are proposed at one month from childbirth, to ensure they have the possibility to participate to all AC meeting. The women answering to this questionnaire represent one third of the entire population of mothers. The authors analysed 1.982 comments given by women attending ACs during the period March–December 2020, corresponding to the period of COVID-19 outbreak, and first and second pandemic waves (almost all women attending somehow an AC or part of this left a comment). The ACs attendance reduced from 64.9% in first quarter to 44.4% in second quarter, to 26.9% in the third quarter, and to 34.7% in the final quarter of 2020.

The second phase of the action research process involved the organisation of meetings and workshops with the key managers and health professionals involved along the maternal and childcare pathway in the Regional Health System (RHS) of the Tuscany Region. While all the mother and child healthcare managers of each LHA were involved, the professionals were identified directly by managers considering their active role in ACs organisation and delivery. Meetings stand for structured encounters where the discussion were managed by the researchers following their presentation, while with workshops the authors mean specific moment of sharing ideas stimulating by a simulation proposed by researchers. Both meetings and workshops were implemented online, due the COVID-19 restrictions. During these organized encounters, the results of the women feedback analysis have been presented, discussed and considered, as evidence for identifying needs, expectations and proposals of women. Researchers facilitated the identification of shared goals with the care provider and support health professionals for facilitating the integration of the organization's resources and the learning processes. Such meetings were aimed at better understanding needs and problems, by sharing the perspectives of users collected by the continuous survey on maternal care pathway, and at discussing the solutions to be implemented in the practice of the ACs provision during the pandemic, by explaining how it was intended to exploit the possibility of integration with the hAPPyMamma system. The achievement of the institutional support to the ACs transformation from all managers of the maternal care pathway was an indirect aim of these meetings, whose main objective was to identify their shared key criteria to follow for this kind of service development.

### Data analysis

For the qualitative data analysis concerning the narratives of women, a mixed inductive and deductive approach was applied in order to develop categories that summarize raw data and convey key themes (Thomas, 2003) and to verify the consistency of the identified categories with the framework used in the study. In particular, we coded the women’s comments using the main broad categories of rooms for innovation, based on their needs and preferences relating to ACs, and their perceived usefulness of innovation solutions, such as online ACs. The results are presented following the key themes emerged, supported by quotes taken from the women's comments themselves. Since the collection of women feedback is continuous, the analysis of their stories has been repeated over time, in order to identify additional information and new inputs.

For the analysis concerning the data from managers’ and health professionals’ involvement, researchers systematized the notes taken during the meetings and workshops, in order to finalize the design of a new AC model. Since the meetings were not video/audio recorded, specific quotations are not available, but the opinions of health operators were categorized and synthetized. The analytical synthesis was based on the framework used in the study and focused on (i) needs, preferences and resistances; (ii) perception of usefulness and ease of use; (iii) identification and standardization of innovation spaces; and (iv) identification of evaluation measures of the innovation impact.

## Results

### Users’ involvement: the women’s point of view on ACs during COVID-19 period

Among the women’s comments analyzed, 517 directly included the terms COVID or emergency (more than 1 out of 4 comments). This shows the pressure that the pandemic had on the maternal care pathway. Two main themes emerged from the analysis of these selected comments: (1) the perceived need of women to attend the ACs that were not carried out or were reduced during the pandemic, and (2) the perceived usefulness of online ACs to answer to this women’s need, which have been reclassified within the first two points of the study framework.

1. Identification of the rooms for innovation in the pathway, by supporting the expression of needs and preferences.

The survey comments of women on ACs focused on the interruption of the face-to-face meetings of ACs starting from March 2020. Many declared how much they were sorry they attended fewer meetings than expected or any at all due to the COVID-19 restrictions, reporting sometimes a feeling to have arrived unprepared for childbirth. Some women expressed regret that the health services were not able to organize alternative forms of ACs and said they felt abandoned by them.

*I only had 3 meetings because everything was cancelled due to COVID… but in my opinion they could continue online or in any case send us information material and instead they abandoned us.*

Alternatively, the choice of women shifted to the autonomous search for information via web, or mainly to the offer of online meetings organised by private professionals, often midwives. There were many comments from women who would have liked the health services to organize online ACs or who complained because online ACs were not offered in their territory compared to others where health professionals had organized themselves spontaneously to have online contacts with users.

*Given the usefulness of this AC, in my opinion it had to be somehow "moved" online. I also compared myself with other women and we missed not being able to finish it. I asked the family care centre and it was explained to me that since ACs is not lectures but also exchange, it would not make sense online. However, for those like me and others who relied on public service in all and for all, it would have been important and reassuring to conclude it precisely online....*

2. Evaluation of specific technological solutions’ perceived usefulness, perceived ease of use.

In the months following the start of the lockdown, the number of comments relating to the use of various communication channels with health personnel (mainly midwives or psychologists) increased: many women reported having had phone calls, used video conferencing systems, or chat applications or received informative materials and videos by email as alternative forms of ACs.

*Online course held by a kind and competent midwife. She managed to reassure us in a particularly delicate period such as that of the lockdown due to the COVID emergency.*

The reported experiences of online meetings during pandemic period are diverse: some women complained about health professionals' unpreparedness or improvisation in this new mode of ACs delivery, but most appreciated attempts to organize online ACs, which they considered useful although they would have preferred face-to-face meetings.

*A very nice course organized online by a midwife due to the COVID emergency that did not allow physically attending an AC.*

Women reported that online ACs served to receive information, have a more concrete insight on childbirth and post-partum, clarify their doubts, share experiences and emotions, not only with health professionals, but also with the other women. The possibility to have online ACs gave to women also the opportunity to feel less alone in the period of isolation due to the health emergency.

*Although it was done via videoconference, it was nice to share our emotions at the time of COVID with other mothers.*

The main criticism emerging from the women’s comments is that online meeting did not always allow the interaction and active participation of those present, considered a key and characterizing aspect of the AC. However, also in this case the experiences seemed to diverge a lot, above all because the ways of organizing the CAs online during the pandemic period were different in local territories. The analysis of women's comments lead to think that online ACs, when properly organized, ensure women the benefits of face-to-face ACs.

*The course was held online due to the COVID-19 emergency. An atmosphere of support and community was created with the other mothers, and I also felt followed, and supported even for the doubts or problems of the first days after giving birth.*

### Services provider involvement: the managers’ and health professionals’ requests for transforming ACs

The research group involved first the managers of maternal and childcare pathway at the regional level and of all the LHAs and THs in Tuscany. Overall, a number of 16 online meetings were organised and put in place in the period May–October 2020 with these institutional stakeholders:

- Two meetings with regional managers;
- Two meetings with the regional committee on maternal care pathway, which is composed by the directors of the maternal care departments at the LHA or TH level;
- Fourteen meetings with the groups of referents for the ACs organisation within each LHA and TH.

In the period October 2020–January 2021, 24 online workshops were realized involving health professionals, mainly midwives, but also psychologists, gynaecologists, prevention doctors and social workers in all the LHA and one TH for an overall amount of around 170 operators. The workshops were used to share the new methods of online provision of ACs and finalise their design, by collecting ideas, suggestions, requests to make the online ACs as appropriate as possible to their needs and those of users.

Through the systematization of the discussions that took place during the meetings and workshops by using the study framework, the following aspects were identified as the most important for managers and health professionals.

1. Identification of the rooms for innovation in the pathway, by supporting the expression of needs and preferences.

From the managers point of view, the homogeneity of the information content conveyed through the ACs to users and of the methods of online provision of ACs was extremely important, as well as they believed integration with the m-health tool already existing at the regional level as strategic. Health professionals, although they considered the ACs in presence the most suitable way, above all stressed the need to identify alternative modalities to carry out them during the COVID-19 emergency, since they considered the participation to ACs very important in the maternal care pathway. The health professionals’ resistance to the use of web technologies to carry out the ACs was therefore overcome by the overwhelmingly felt need to continue to maintain this service for users.

2. Evaluation of specific technological solutions’ perceived usefulness, perceived ease of use, and related habits of professionals to be changed

The reflections and remarks on the type of technological solution to be adopted arose mainly from the discussion with the health professionals and managers most directly involved in the ACs organization, who better had in mind the needs to be met in identifying the new solution. In particular, they appreciated the proposal of the web platform integrated in the hAPPyMamma system where sharing materials and carrying out online ACs for the perceived usefulness and ease of use of this digital solution and the simplification of the organization and management of the online ACs compared to the solutions identified extemporaneously in the first period of the pandemic. One of the most relevant aspects was the possibility of using the information recorded in the hAPPyMamma system to be able to organize the ACs and contact users, thus being able to offer this service more easily even in a proactive manner to all potential participants. Moreover, they particularly valued the flexibility of the digital solution management to customize the contents and methods of interaction with users, based on the specificity of the local organisation, although within a uniform framework of the online ACs. However, during the workshops with health professionals, their concern about having to use technological tools they had little mastery also emerged. Even the use of videoconferencing system itself to carry out the online ACs was of particular concern to older health professionals and, in general, everyone observed that the proposed innovation has an impact on their practice and requires an effort to adapt their own habits as professionals.

3. Identification of equitable and long-lasting innovative solutions, to better responding to both providers and users’ needs during and after the crisis

According to the point of view of health professionals, a key characteristic that ACs should maintain also in the online way of delivery was the possibility to interact actively with the group of women during online meetings. Users as well considered crucial this aspect, which emerged as one of the main factors of success of the online ACs. The collaborative approach allowed setting the use of web tools as much interactive as possible. However, researchers tried to share with managers, health professionals and users that this aspect may be affected by infrastructural factors (mainly web connection and quality of devices used), which constitute essential prerequisites for the sustainability of the innovation introduced. The long-term perspective shared by managers and health professionals was that even where the ACs return to being held in person, the web tools made available could continue to be used and three main scenarios have been identified:

- Maintenance of some ACs organised entirely online in order to involve users who have difficulties to participate in presence for reasons of health, work or travel problems;
- Organisation of ACs in presence that have some online meetings;
- Organisation of ACs entirely in presence but continuing to use the platform's web tools as further information and communication channels.

The shared idea was that the opportunity opened by COVID-19 must be maintained even later, perhaps re-adapting it to the conditions that would arise later. In particular, it was highlighted during the workshops that online ACs could be an effective and equitable way to involve groups of foreign women, who have more difficulty in participating in traditional ACs. Indeed, this innovative solution in healthcare provision could facilitate the organization of online ACs involving groups of women with the same citizenship who perhaps live in larger territories with the support of linguistic-cultural mediation.

4. Identification of indicators to measure the innovation’s and transformations’ impact

A last point that was discussed mainly during the meeting with managers regarded the monitoring and evaluation of online ACs, focussing in measuring participation and perceived quality thanks to the administrative data from health services and quantitative and qualitative data collected by the continuous survey on the experience of users within the maternal care pathway. In particular, managers and professionals agreed on using the following key indicators to monitor the online ACs implementation and measure the impact of this innovative solution:

- ACs attendance;
- Level of discussion of different topics concerning maternal and childcare pathway during the ACs;
- Perceived usefulness of ACs.

Moreover, periodic meetings with health professionals were planned in order to collect also their feedback on the experience of carrying out online ACs, identifying pros and cons of this innovative solution and aspects to be improved.

### Co-innovation of ACs: design and implementation

As the result of the combination of the two components of the action-research process involving users and health professionals, in between December 2020 and January 2021, a final version of the online ACs model was released and approved by all the involved parties. The individual healthcare professionals or organizations that autonomously developed some ‘home-made’ solutions moved to the regional model for providing online ACs. However, the research group is aware that the progressive implementation of the online ACs in each territory could bring indications for further changes in the model, as feedback from users and health professionals could identify needs to improve the implemented online CAN model, in line with the value co-creation approach.

The co-created online ACs design presented the key following characteristics:

- Provision of a management tool for the creation of groups of users to be invited to participate in ACs starting from the information contained in the hAPPyMamma system;
- Provision of a virtual space for users where they can find multimedia information materials and tools for synchronous and asynchronous communication with health professionals, corresponding to different organisational level (Region, LHA, TH, districts);
- Access to this virtual space from app hAPPyMamma in order to facilitate the users’ experience and give then an integrated view of the resources for the maternal care pathway;
- Creation and publication of videos in this virtual space that facilitate the use of information on the maternal care pathway, together with further information materials (documents, brochure, poster);
- Use of tools for interaction, specifically virtual classroom to meet group of users and forum for asynchronous interaction with them.

In this initial phase of the online ACs implementation, the hypothesis of organizing meetings dedicated to groups of women with the same citizenship with the support of linguistic-cultural mediation was not carried out.

Starting from mid-December 2020, the health professionals have gradually started to organize online ACs. At the end of the first semester 2021, in 22 out of 26 districts of the LHA and one TH online ACs have been delivered with the involvement mainly of midwives, but also psychologists, social workers, prevention doctors, paediatricians, and around 5000 users. Monitoring the ongoing survey data on the maternal care pathway, the ACs attendance started to increase again reaching 54.9% at the end of the first semester 2021. The level of discussion of different topics that the women reported to have experience during the ACs was slightly minor of that reported by the women in pre-pandemic period, when the number of AC meetings was greater, but it was almost coherent in all post-pandemic period. The theme concerning labour and delivery maintained the same levels of discussion as before the COVID-19, with the only exception of the initial 2 months of the outbreak, when this level had inevitably decreased also for this topic. The perceived utility of ACs shows a similar trend, with a slight decline after the COVID-19 outbreak and a maintenance later. The main critical aspects mentioned by women on the online ACs concerns the problems due to web connection that impact negatively on their perceived quality. Moreover, the activities on the body, such as breathing exercises, which were done in the face-to-face meetings and were appreciated by women, were not almost done in the online ACs.

In the last part of the first semester 2021 in correspondence with the relaxation of restrictions due to pandemic, even in the areas where face-to-face ACs have been resumed, the online tools made available for identifying groups of users, sharing multimedia materials on maternal care pathway and communicating with users through asynchronous interaction have been continuing to be used. During the monitoring meetings of the online ACs implementation, managers and health professionals agreed that this solution could be integrated with the traditional model in presence also in post-pandemic period. The integration could be done through the organization of ACs with some face-to-face meetings and others online.

## Discussion

The study described an action-research process that supported health organizations to answer to the need—emerged with the COVID-19 pandemic—of identifying innovative solutions in the health service provision, by facilitating this innovation design and implementation through a collaborative approach. The study pointed out that an approach based on the involvement of managers, health professionals and users in co-assessment of needs and preferences and co-design technological solutions suitable for addressing these needs and preferences, resulted an enabling factor of successful, long-lasting and integrated solutions in healthcare, specifically for ACs in the maternal care pathway. Indeed, the involvement of users and providers is essential for the value co-creation in healthcare, particularly for new service developments and for innovating still existing services by using existing resources to co-create value (Edvardsson et al., 2014; Hau et al., 2017; Vargo et al., 2020). Users and healthcare professionals are not only considered potential sources of knowledge and innovation by the health organization (Lush & Vargo, 2008), but are key actors in the process of service innovation and change. This kind of interaction intrinsically resulted a win–win process, in which the service provider has the opportunity to improve its services, and users can benefit from the adjusted or customised services in response to their characteristics, needs and preferences, thus arriving to enhance the experiential value they perceived and their satisfaction (Grönroos, 2008; Osei-Frimpong, 2016; Lush et al., 2007). By applying this approach, the researchers supported the public healthcare system in take advantage of a crisis for defining a new way to provide the ACs in the maternal care pathway.

The deletion of ACs due to COVID-19 pandemic represents a pressure from the external environment imposed on healthcare organizations to change the services provision model (Wickramasinghe & Schaffer, 2010). This factor facilitated the adoption of a new approach that moved ACs on the web, by overcoming organizational and human barriers and resistance to changes that previously had prevented healthcare organizations from thinking about the implementation of this solution (Lauria et al., 2012). In the COVID-19 period, the perceived usefulness of the online ACs was greater of these resistances. The critical circumstances produced by the pandemic led professionals to adapt and introduce new ways of providing services in the maternal care pathway, in particular ACs. This produced a patchy implementation, as directly reported by the users, and accordingly to other experiences of innovation in the Italian healthcare system (Petracca et al., 2020). Instead, the co-creation process that has been supported and analysed in this study led to the implementation of a standardized and long-lasting online ACs, by involving the practitioners in a reflective and professional self-development process. The co-production process ensured that the users’ point of view served as change-driver, and that clear and shared standard were met in the innovation design and implementation with managers and professionals. Healthcare organizations adhered to the common online ACs design, thus enjoining a regional model. This decreased the initial fragmentation of the approaches to the web transition of the ACs. The value co-creation process generated innovation, through dialogue, participation and empowerment of all actors involved: users, health professionals, and researchers. This happened within a social, collaborative and creative process allowing stakeholders’' empowered engagement and creativity to generate insights and latent opportunities, not only for the design of services, but also to their production and continuous development. In particular, the feedback of users has been collected, analysed and discussed with practitioners (Elg et al., 2012), who paid attention to women opinions about their satisfaction with the service (Berry & Bendapudi, 2007). In this way, healthcare users have been explicitly, although indirectly, involved in the co-design process, by a co-assessment process. Their feedback informed practitioners with their knowledge and capabilities with the aim to improve healthcare service (Russo et al, 2019).

The study showed how relevant has been moving ACs on the web, since the need of pregnant women to share information and receive emotional support was accentuated in times of crisis, as emerged from the analysis of users’ comments collected with the continuous survey. Finding the innovative solution of the online ACs allowed users to continue to participate in ACs, as showed by the ACs attendance rate over the pandemic period that restart increasing thanks to the online ACs implementation, thus avoiding depriving them of ACs’ potential value in terms of health benefits for themselves and their child. Indeed, previous researches proved that the participation to ACs positively affects health outcomes for mothers and children, retention in the use of family care services in the post-natal period, satisfaction in the maternal care pathway (Grussu & Quataro, 2003; Murante et al., 2014). Moreover, participation to ACs allows early interventions of professionals in preventing postpartum depression (Kerber et al., 2007), and in promoting and developing health potentials of children in the first period their life, according to the life course approach (Bonciani & Lupi, 2017) inspiring the Ministry of Health guidelines on the first thousand days. The monitoring and evaluation measures that have been identified with managers, during the action-research process, will contribute to verify over the time whether the online ACs would confirm these positive benefits, such as better experience and health outcomes reported by women participating to the online ACs. The expected results shared by the research group are that the innovative model of ACs provision would not diminish the proven benefits for women, provided that the online ACs maintain key characteristics considered crucial by users and professionals, such as a good level of interaction. Therefore, it will have to be verified whether this aspect, which was clearly pointed out in the co-assessment and was included in the online ACs co-design, was then actually implemented, or whether obstacles that do not concern the development of the online ACs itself (such as shortcomings in the connections used) limited in fact the interaction. Indeed, other studies showed that innovations introduced quickly in the Italian healthcare, due to and thanks to COVID-19 emergency, had to face an unprepared context, especially from the point of view of infrastructures for digitization (Petracca et al., 2020). The availability of an observatory on the women experience with maternal care service, based on the continuous longitudinal web survey implemented in Tuscany Region, is a key enabler of the self-reflective spiral of cycles of observation, reflection and planning, implementation of action as the intervention, evaluation and moving to the next stage of planning, which characterise the action-research process. Thus, in terms of opportunities for further research, with regards to other data sources, the results from the continuous survey on the maternal care pathway may be used to evaluate the change in the women’s experience, in COVID-19 period with the deletion of ACs and after the introduction of the online ACs. The data on the level of discussion of different topics concerning maternal and childcare pathway and the perceived usefulness of ACs showed a small reduction compared to the pre-pandemic period and it will be important to investigate the reasons that determined them. In particular it will be necessary understand how much these perceptions have been influenced by external factors, such as the limitations of the web connection quality, or by internal factors, such as the shorter duration, or the lack of activity on the body, or less competence of health personnel to carry out online meetings effectively. Indeed, the authors planned to carry out a specific web-based survey addressing women attending online ACs to assess their perception on the web ACs’ quality and effectiveness and identify aspects of online ACs that could be improved, thanks to this continuous co-assessment carried out by women.

Finally, the new ACs provided online seemed to be a valuable solution not only for the contingent situation due to COVID-19 emergency, but also in a long-term perspective, to reach more women during pregnancy and to early support them throughout the maternal care pathway. Indeed, even before the health emergency, the most fragile cases (i.e., less educated women, foreigners or women with difficulties in reconciling work and ACs) had difficulties to attend ACs (Bonciani et al., 2020). In the future, the potential of this provision of health services could contribute an equal access to ACs. In this way, the innovative solution for providing ACs that was developed with the collaborative process could be considered a valuable way for decreasing some inequalities, both those existing before pandemic, and those produced by the pandemic itself (Tarricone & Rognoni, 2020). Although the most of healthcare professionals expressed interest to come back the traditional way of ACs provision, further modalities of articulation of the service provision model would be implemented in future, following the logic of complementarity of different combinations of service provision factors. The online ACs could be maintained after the end of pandemic for the groups of women that prefer this channel, while the traditional ACs could be available for other groups of women. Additionally, other forms of integrated forms of ACs could be created, such as mixed ACs with face-to-face and online meetings, using the tools made available thanks to this intervention. To avoid the risk to simply fall back to status quo ante in a post-crisis period, as highlighted by other authors (Bashshur et al. 2020), it will especially matter how much the ‘perceived usefulness’ of this innovative solution implemented during the COVID-19 emergency will be notified by managers, health professionals, but also users.

This research contributes to the literature in different ways.

First, our results confirms that the co-production processes is a relevant enabling factors in the innovation design, acceptance, and implementation. Healthcare managers and professionals involved into the study used the women insights and suggestions as a key confirmation of the usefulness of the technological innovation of ACs. The collaborative process facilitated the standardization of approaches to the online transition of ACs, by providing a common and agreed model toward which every healthcare organization in Tuscany moved. The various individual and often ‘hand-made’ solutions were substituted by the regional online ACs models, thus overcoming fragmentation, and providing an innovative solution that can survive the pandemic.

Second, this research used a multi-stakeholder methodology, by giving a critical role to scholars and researchers, involving indirectly a very high number of women with a permanent survey (data from more than 4700 women in the overall period since pre-COVID-19 outbreak until the first semester 2021), all healthcare managers of the Tuscan maternal-pathway and around 170 healthcare professionals. This is a quite important and rare aspect in an action-research, which makes robust and interesting the real-word evidence that support the hypothesis of the importance of multi-stakeholder collaborative processes in innovating healthcare.

Third, there is scares marketing literature focussing on value co-creation in healthcare during the COVID-19 pandemic, and focussing on the innovation processes. The paper’s originality is represented by the analysis of how a critical situation, such as COVID-19, can become an opportunity for healthcare organizations to overcome barriers to innovation, pushing the co-design of standard and shared innovative model at a regional level, and using a collaborative process to provide new e-health/m-health solutions that improve the user’s experience.

In sum, from a theoretical point of view, our study extends knowledge on value co-creation in healthcare by demonstrating the central role of multi-stakeholder collaborative processes in designing and introducing standard and potentially long-lasting innovations. Results show how external and internal stakeholders could be more widely involved into the innovation design and implementation, by considering their needs, perception and, thus, increasing the acceptability of the innovation itself.

## Conclusion

The action research presented in this paper describes the strategy adopted by the healthcare organizations during the COVID-19 pandemic for dealing with a practical problem (the need to provide ACs) and identifying an innovative solution that generated possibilities for change in healthcare (ACs via web) with the collaboration of all stakeholders. The study contributes to confirm according to the theoretical framework that the collaborative approach adopted is an enabling factor of successful, long lasting and integrate innovation in healthcare.

Despite the nature of the action-research is context-specific, the findings presented in this paper have also practical implications for other healthcare organizations and systems. Indeed, this experience can help other healthcare organizations to innovate their own strategies in ACs’ provision. The involvement of both providers and users of healthcare services in co-assessing the services and co-innovating them represented a key aspect of success of this experience. In fact, it allowed co-producing an innovation moving from single patchy experiences to a standard solution based on the integration of users’ and professionals’ resources.

## Declarations

### Data availability

The material is available upon direct request to the authors of the manuscript.

## References

[CR1] Abell DF (1978). Strategic windows: The time to invest in a product or market is when a ‘strategic window’is open. Journal of Marketing.

[CR4] Baker GR, Denis JL (2011). Medical leadership in health care systems: From professional authority to organizational leadership. Public Money & Management.

[CR5] Bashshur R, Doarn CR, Frenk JM, Kvedar JX, Woolliscroft JO (2020). Telemedicine and the COVID-19 pandemic, lessons for the future. Telemed e-Health.

[CR6] Batalden M, Batalden P, Margolis P, Seid M, Armstrong G, Opipari-Arrigan L, Hartung H (2016). Coproduction of healthcare service. BMJ Quality and Safety.

[CR7] Beirão G, Patrício L, Fisk RP (2017). Value cocreation in service ecosystems: investigating health care at the micro, meso, and macro levels. Journal of Service Management.

[CR002] Belso-Martínez JA, Mas-Tur A, Sánchez M, López-Sánchez MJ (2020). The COVID-19 response system and collective social service provision. Strategic network dimensions and proximity considerations. Service Business.

[CR8] Berry LL (2019). Service innovation is urgent in healthcare. AMS Review.

[CR9] Berry LL, Bendapudi N (2007). Health care: A fertile field for service research. Journal of Service Research.

[CR001] Bidmead E, Marshall A (2020). Covid-19 and the “new normal”: are remote video consultations here to stay?. British Medical Bulletin.

[CR10] Bonciani, M., Lupi, B. (2017). Monitoraggio dell’allattamento materno in Toscana. Prima parte. Report 2016–2017.

[CR11] Bonciani M, Corazza I, Lupi B, De Rosis S (2020). How to improve the maternal pathway for migrant women: Insights for retention strategies from tuscany region. Micro & Macro Marketing.

[CR12] Bonciani M, De Rosis S, Vainieri M (2021). Mobile health intervention in the maternal care pathway: Protocol for the impact evaluation of hAPPyMamma. JMIR Research Protocols.

[CR13] Bosa I, Castelli A, Castelli M, Ciani O, Compagni A, Galizzi MM, Garofano M, Ghislandi S, Giannoni M, Marini G, Vainieri M (2021). Response to COVID-19: Was Italy (un)prepared?. Health Economics, Policy and Law.

[CR012] Brandsen T, Pestoff V (2006). Co-production, the third sector and the delivery of public services. Public Management Review.

[CR008] Carlborg P, Kindström D, Kowalkowski C (2014). The evolution of service innovation research: a critical review and synthesis. The Service Industries Journal.

[CR14] Chen SH, Wen PC, Yang CK (2014). Business concepts of systemic service innovations in e-Healthcare. Technovation.

[CR15] Chowdhury, A., Hafeez-Baig, A., Gururajan, R., & Chakraborty, S. (2019). Conceptual framework for telehealth adoption in Indian healthcare. In *24th Annual Conference of the Asia Pacific Decision Sciences Institute: Full papers*. Asia-Pacific Decision Sciences Institute (APDSI).

[CR007] Cui AS, Wu F (2016). Utilizing customer knowledge in innovation: antecedents and impact of customer involvement on new product outcomes. Journal of Academy of Marketing Science.

[CR17] Davis, F. D. (1989). Perceived usefulness, perceived ease of use, and user acceptance of information technology. *MIS Quarterly*, 319–340.

[CR18] De Rosis S, Barsanti S (2017). Multi-level determinants of tele-healthcare: A case study in Italy. International Journal of Healthcare Technology and Management.

[CR19] De Rosis S, Nuti S (2018). Public strategies for improving eHealth integration and long-term sustainability in public health care systems: Findings from an Italian case study. The International Journal of Health Planning and Management.

[CR20] De Rosis S, Pennucci F, Noto G, Nuti S (2020). Healthy living and co-production: Evaluation of processes and outcomes of a health promotion initiative co-produced with adolescents. International Journal of Environmental Research and Public Health.

[CR21] De Rosis S, Pennucci F, Nuti S (2019). From experience and outcome measurement to the health professionals’ engagement. Micro & Macro Marketing.

[CR22] Downer T, McMurray A, Young J (2020). The role of antenatal education in promoting maternal and family health literacy. International Journal of Childbirth.

[CR23] Edvardsson B, Gustafsson A, Kristensson P, Tronvoll B, Witell L, Rust RT, Hung M-H (2014). New service development from the perspective of value co-creation in a service system. Handbook on research in service marketing.

[CR24] Elg M, Engström J, Witell L, Poksinska BB (2012). Co-creation and learning in health-care service development. Journal of Service Management..

[CR005] Etgar M (2008). A descriptive model of the consumer co-production process. Journal of the Academy of Marketing Science.

[CR26] Gray M, Pitini E, Kelley T, Bacon N (2017). Managing population healthcare. Journal of the Royal Society of Medicine.

[CR27] Grönroos C (2008). Service logic revisited: who creates value? And who co-creates?. European Business Review.

[CR28] Grussu, P., & Quataro, R. M. (2003). Corsi pre-parto e rilevazione precoce della depressione postnatale. *Psicologia Della Salute*.

[CR29] Gummesson E (2000). Qualitative methods in management research.

[CR30] Hashiguchi, T.O. (2020). Bringing health care to the patient: an overview of the use of telemedicine in OECD countries. OECD Health Working Papers, No. 116. Paris: OECD Publishing. 10.1787/8e56ede7-en.

[CR31] Hau L, Anh P, Thuy P (2017). The effects of interaction behaviors of service frontliners on customer participation in the value co-creation: A study of health care service. Service Business.

[CR003] Heinonen K, Strandvik T (2021). Reframing service innovation: COVID-19 as a catalyst for imposed service innovation. Journal of Service Management.

[CR32] Hendy J, Barlow J (2012). The role of the organizational champion in achieving health system change. Social Science & Medicine.

[CR006] Hienerth C, Lettl C, Keinz P (2014). Synergies among producer firms, lead users, and user communities: the case of the LEGO producer–user ecosystems. Journal of Product Innovation Management.

[CR33] Hougaard S (2006). The business idea: The early stages of entrepreneurship.

[CR34] Joiner K, Lusch R (2016). Evolving to a new service-dominant logic for health care. Innovation and Entrepreneurship in Health.

[CR35] Karazivan P, Dumez V, Flora L, Pomey MP, Del Grande C, Ghadiri DP, Fernandez N, Jouet E, Las Vergnas O, Lebel P (2015). The patient-as-partner approach in health care: A conceptual framework for a necessary transition. Academic Medicine.

[CR36] Kellermann AL, Jones SS (2013). What it will take to achieve the as-yet-unfulfilled promises of health information technology. Health Affairs.

[CR37] Kerber KJ, de Graft-Johnson JE, Bhutta ZA, Okong P, Starrs A, Lawn JE (2007). Continuum of care for maternal, newborn, and child health: From slogan to service delivery. The Lancet.

[CR38] Kierkegaard P (2015). Mapping telemedicine efforts: Surveying regional initiatives in Denmark. Telemedicine and e-Health.

[CR39] Lauria, L., Lamberti, A., Buoncristiano, M., Bonciani, M., & Andreozzi, S. (2012). Percorso nascita: Promozione e valutazione della qualità di modelli operativi. Le indagini del 2008–2009 e del 2010–2011. Rapporto Istisan 12/39.

[CR41] Lee D (2019). Effects of key value co-creation elements in the healthcare system: Focusing on technology applications. Service Business.

[CR42] Liew EJY, Koh SGM, Andrei OJK, Poh YH, French JA, Han HS, Lee J (2020). Technology perception and productivity among physicians in the new norm post-pandimic: a dynamic ca- pabilities perspective. COVID-19 and the future of the service industry post-pandemic: Insights and resources.

[CR43] Lusch RF, Vargo SL, O’brien M (2007). Competing through service: Insights from service-dominant logic. Journal of Retailing.

[CR44] Mintzberg H, Asch D, Bowman C (1989). The structuring of organizations. Readings in strategic management.

[CR45] Mosley D (2014). Supervisory management.

[CR46] Murante AM, Nuti S, Matarrese D (2014). The maternity pathway report.

[CR47] Nabatchi T, Sancino A, Sicilia M (2017). Varieties of participation in public services: The who, when, and what of coproduction. Public Administration Review.

[CR009] Nguyen HN, Rintamäki T, Saarijärvi H, Smedlund A, Lindblom A, Mitronen L (2018). Customer value in the sharing economy platform: the Airbnb case. Collaborative value co-creation in the platform economy.

[CR011] Norman DA, Carroll JM (1991). Cognitive artifacts. Designing interaction: psychology at the human-computer interface.

[CR015] Normann R (2001). Reframing business: when the map changes the landscape.

[CR48] Nuti, S., Bini, B., Ruggieri, T. G., Piaggesi, A., & Ricci, L. (2016a). Bridging the gap between theory and practice in integrated care: The case of the diabetic foot pathway in Tuscany. *International Journal of Integrated Care*, *16*(2). 10.5334/ijic.1991 PMC5356204 29042842

[CR49] Nuti S, Vola F, Bonini A, Vainieri M (2016). Making governance work in the health care sector: Evidence from a ‘natural experiment’ in Italy. Health Economics, Policy and Law.

[CR50] Ollila S, Yström A (2020). Action research for innovation management: Three benefits, three challenges, and three spaces. R&D Management.

[CR51] Osborne SP, Radnor Z, Strokosch K (2016). Co-production and Co-creation of value in public services: A suitable case for treatment. Public Management Review.

[CR52] Osei-Frimpong, K. (2016). Examining the effects of patient characteristics and prior value needs on the patient-doctor encounter process in healthcare service delivery. *International Journal of Pharmaceutical and Healthcare Marketing*.

[CR019] Osei-Frimpong K, Wilson A, Owusu-Frimpong N (2015). Service experiences and dyadic value co-creation in healthcare service delivery: a CIT approach. Journal of Service Theory and Practice.

[CR016] Ostrom E (1996). Crossing the great divide: coproduction, synergy, and development. World Development.

[CR53] Patrício L, GrenhaTeixeira J, Vink J (2019). A service design approach to healthcare innovation: From decision-making to sense-making and institutional change. AMS Review.

[CR017] Pestoff V (2012). Co-production and third sector social services in Europe: some concepts and evidence. Voluntas.

[CR54] Petracca F, Ciani O, Cucciniello M, Tarricone R (2020). Harnessing digital health technologies during and after the COVID-19 pandemic: context matters. Journal of Medical Internet Research.

[CR004] Prahalad CK, Ramaswamy V (2004). Co-creating unique value with customers. Strategy & Leadership.

[CR020] Renkert S, Nutbeam D (2001). Opportunities to improve maternal health literacy through antenatal education: an exploratory study. Health Promotion International.

[CR55] Russo G, MorettaTartaglione A, Cavacece Y (2019). Empowering patients to co-create a sustainable healthcare value. Sustainability.

[CR57] Sebastiani R, Anzivino A, Han HS, Lee J (2020). Transformative value co-creation in healthcare services in the COVID-19 Era. COVID-19 and the future of the service industry post-pandemic: Insights and resources.

[CR013] Sorrentino M, Guglielmetti C, Gilardi S, Marsilio M (2015). Health care services and the coproduction puzzle: filling in the blanks. Administration & Society.

[CR58] Spinelli A, Baglio G, Donati S, Grandolfo ME, Osborn J (2003). Do antenatal classes benefit the mother and her baby?. The Journal of Maternal-Fetal & Neonatal Medicine.

[CR59] Sweeney JC, Danaher TS, McColl-Kennedy JR (2015). Customer effort in value cocreation activities: Improving quality of life and behavioural intentions of health care customers. Journal of Service Research.

[CR60] Tanna NK (2005). Action research: A valuable research technique for service delivery development. Pharmacy World and Science.

[CR61] Tarricone R, Rognoni C (2020). What can health systems learn from COVID-19?. European Heart Journal Supplements: Journal of the European Society of Cardiology.

[CR021] Thomas DR (2003). A general inductive approach for qualitative data analysis.

[CR62] Tjosvold D, MacPherson RC (1996). Joint hospital management by physicians and nursing administrators. Health Care Management Review.

[CR010] Trenz M, Frey A, Veit D (2018). Disentangling the facets of sharing: a categorization of what we know and don’t know about the sharing economy. Internet Research.

[CR64] Van de Ven AH, Johnson PE (2006). Knowledge for theory and practice. The Academy of Management Review.

[CR65] Vargo SL, Akaka MA, Wieland H (2020). Rethinking the process of diffusion in innovation: A service-ecosystems and institutional perspective. Journal of Business Research.

[CR66] Vargo S, Lusch R (2008). Service-dominant logic: Continuing the evolution. Journal of the Academy of Marketing Science.

[CR67] Venkatesh V, Davis FD (2000). A theoretical extension of the technology acceptance model: Four longitudinal field studies. Management Science.

[CR68] Venkatesh V, Thong JY, Chan FK, Hu PJH, Brown SA (2011). Extending the two-stage information systems continuance model: Incorporating UTAUT predictors and the role of context. Information Systems Journal.

[CR018] Virlée J, van Riel ACR, Hammedi W (2020). Health literacy and its effects on well-being: how vulnerable healthcare service users integrate online resources. Journal of Services Marketing.

[CR014] Voorberg WH, Bekkers VJJM, Tummers LG (2015). A systematic review of co-creation and co-production: embarking on the social innovation journey. Public Management Review.

[CR69] WHO 2021. https://www.euro.who.int/en/data-and-evidence/evidence-informed-policy-making/publications/pre2009/what-is-the-efficacyeffectiveness-of-antenatal-care#:~:text=The%20purpose%20of%20antenatal%20care,and%20birth%20as%20positive%20experiences.

[CR71] Wickramasinghe N, Schaffer J (2010). Realizing value driven e-health solutions.

[CR72] Woolliscroft JO (2020). Innovation in response to the COVID-19 pandemic crisis. Academic Medicine: Journal of the Association of American Medical Colleges.

[CR73] Yin RK (1998). The abridged version of case study research. Handbook of Applied Social Research Methods.

[CR74] Zainuddin N, Russell-Bennett R, Previte J (2013). The value of health and wellbeing: an empirical model of value creation in social marketing. European Journal of Marketing.

